# Optimising Psychological Well-Being in Chinese-Australian Adolescents: A 24-Hour Movement Guidelines Approach

**DOI:** 10.3390/children12030329

**Published:** 2025-03-05

**Authors:** Wei-Cheng Chao, Asaduzzaman Khan, Jui-Chi Shih, Wen Li, Ching-Lin Wu, Kuan-Chung Chen, Bill Cheng

**Affiliations:** 1 Program in Tissue Engineering and Regenerative Medicine, College of Medicine, National Chung Hsing University, 145 Xingda Road, South District, Taichung 402, Taiwan; m9506015@gmail.com (W.-C.C.); a14651@show.org.tw (K.-C.C.); 2 Department of Orthopedics, Show Chwan Memorial Hospital, No. 542, Section 1, Zhongshan Road, Changhua 500, Taiwan; 3 School of Health and Rehabilitation Sciences, The University of Queensland, St. Lucia, QLD 4072, Australia; akhan2@uq.edu.au; 4 Graduate Institute of Sports and Health Management, National Chung Hsing University, 145 Xingda Road, South District, Taichung 402, Taiwan; s00382872@myacu.edu.au (J.-C.S.); psclw@dragon.nchu.edu.tw (C.-L.W.); 5 School of Education, The University of Queensland, St. Lucia, QLD 4072, Australia; wen.li@uq.edu.au; 6 Graduate Institute of Biomedical Engineering, National Chung Hsing University, 145 Xingda Road, South District, Taichung 402, Taiwan

**Keywords:** 24-h movement guidelines, physical activity, screen time, sleep, well-being, adolescents

## Abstract

Background: Chinese-Australian adolescents face unique academic and cultural challenges that may impact their lifestyle and psychological well-being. Physical activity, screen time, and sleep are known to influence well-being. However, research on the adherence to the 24-Hour Movement Guidelines among Chinese-Australian adolescents remains limited and awaits further investigation. Objective: This study hypothesized a significant positive association between adherence to the 24-Hour Movement Guidelines for physical activity, screen time, and sleep, and the psychological well-being of Chinese-Australian adolescents. Methods: A self-reported questionnaire was distributed to two language schools in Brisbane, Australia, targeting high school students from grades 7 to 12 with Chinese-Australian backgrounds. This study used multiple linear regression modelling to examine the associations between meeting or not meeting recommendations. Meeting the 24-Hour Movement Guidelines was defined as ≥60 min/day of moderate to vigorous physical activity (MVPA), ≤2 h/day of recreational screen time, and 9–11 h/night of sleep. Results: Out of 251 participants (average age: 13.31 years; 58% female), only 20.3% met two or three recommendations, while 43.3% met one, and 36.2% met none. The most common compliance was meeting only the screen time guideline alone (48%), while 9.6% met either MVPA + screen time or screen time + sleep. The regression analysis showed that meeting at least MVPA (β = 1.41, 95% CI: 0.07 to 2.74) or at least sleep (β = 1.40, 95% CI: 0.19 to 2.60) was associated with better psychological well-being. Notably, meeting MVPA and sleep guidelines was significantly associated with higher well-being (β = 3.83, 95% CI: 1.06–6.60). From the results, adherence to additional 24-Hour Movement Guidelines was associated with improved psychosocial well-being. However, a small proportion of adolescents met all the guidelines. Conclusions: Greater adherence to physical activity and sleep guidelines is linked to better psychological well-being among Chinese-Australian adolescents. These results highlight the importance of promoting healthy behaviours and implementing public health strategies to enhance education on exercise and sleep, particularly at the school and family levels, to support adolescents’ psychological well-being.

## 1. Introduction

Renowned for its cultural and linguistic diversity (CALD), Australia boasts a thriving Chinese community exceeding 1.3 million strong (5.5% of the population). The adolescents in this community often face high academic pressure, especially considering the high emphasis that Chinese families place on education [1,2]. To some extent, such academic pressure drives adolescents to devote a significant amount of time to studying and using electronic devices. This leads to increased sedentary behaviour and negatively impacts physical activity and sleep duration [3]. This focus on achievement and goal-setting seems to benefit math performance and engagement, boosting competence and aspirations. However, as adolescents enter high school, academic pressure rises, leading to increased screen time and sedentary behaviour, while exercise and sleep decrease. Long-term physical inactivity can negatively affect brain health and emotional stability, making it a major focus of current research. These changes in health behaviours are closely related to various psychological issues, including anxiety, depression, and even an increased risk of suicide [4]. Previous research has shown that physical activity, sleep, and sedentary behaviour play essential roles in improving adolescent psychological well-being, effectively reducing depression and anxiety, and enhancing self-esteem [5,6].

Adolescence is a crucial time to build healthy habits. Recognising this, Canada and the WHO championed the concept of 24 h movement guidelines (24-HMGs) for children and youth. The 24-HMGs provide recommendations on physical activity, screen time, and sleep, and have been proven to play a positive role in improving both the physical and psychological well-being of adolescents [7]. Board research has demonstrated a direct association between adherence to these recommendations and improved health outcomes. Within a 24 h period, the combined effects of sleep, sedentary time, light physical activity (LPA), and moderate-to-vigorous physical activity (MVPA) significantly influence the physical, social, and psychological well-being of children and adolescents [8]. Compliance with these recommendations for movement guidelines has been linked to improvements in health-related quality of life (HRQoL), showing better outcomes in both physical health and psychosocial well-being [9]. This research highlights the importance of each guideline component in promoting overall well-being. Several studies have focused on Australian adolescents’ adherence to the 24-HMGs [9,10]. Previous research has also examined the prevalence of physical inactivity and excessive screen time among Chinese adolescents [5].

In response to growing concerns over adolescent sedentary behaviour and screen time, the Australian government updated the 24-Hour Movement Guidelines (24-HMGs) in 2021 [11]. Research consistently links excessive screen time and physical inactivity to various health issues, including weight gain, poor diet, anxiety, attention difficulties, and low self-esteem [12,13,14]. These behaviours tend to increase during the transition to secondary school, driven by academic pressures and screen-based leisure activities [10], with prolonged screen exposure also disrupting sleep patterns [15]. To address these risks, the 24-HMGs recommend limiting recreational screen time to two hours per day [11]. Regular physical activity plays a vital role in adolescent well-being, helping to reduce depression and anxiety while improving self-esteem and cognitive function [16]. Studies indicate that greater physical activity and reduced screen time in younger teens are associated with fewer depressive symptoms [17], reinforcing the 24-HMG recommendation of at least 60 min of moderate-to-vigorous physical activity (MVPA) daily for optimal health [11]. Equally important, sleep is a critical factor in overall well-being, with insufficient rest linked to a 55% increase in the likelihood of mood deficits [18]. However, adolescents today sleep less than previous generations, which negatively impacts academic performance, health, and daily functioning. To promote healthy sleep habits, the National Sleep Foundation recommends 9–11 h of sleep per night for children (6–13 years) and 8–10 h for adolescents (14–17 years) [19].

While numerous studies examine the impact of exercise, screen time, and sleep on adolescent health, research specifically focused on Chinese-Australian adolescents remains limited. Under the pressure of academic and family expectations, these teenagers often struggle to meet recommended guidelines for physical activity, sleep, and screen time. However, the relationship between these behaviours and their psychological well-being, particularly within a cultural context, has not been systematically explored. This study aims to bridge this gap by analysing compliance with the 24-HMGs among Chinese-Australian adolescents and assessing how adherence influences psychological health. We hypothesize that meeting a greater number of 24-HMG recommendations—covering exercise, sleep, and screen time—will be associated with improved psychological well-being. The findings of this study will provide targeted preventive measures and practical guidance for reducing the risk of depression and anxiety while enhancing adolescent physical health. Furthermore, the results will offer valuable insights for public health interventions, supporting the development of culturally tailored strategies to promote healthier lifestyles among Chinese-Australian youth.

## 2. Material and Methods

Regarding sample size, within-subject correlation values typically require a certain sample size to achieve 80% power [20]. Using the bootstrap method, a sample size of approximately 300 was required when the mediation effect size was medium, the within-subject correlation was high (ICC = 0.8), and three repeated measures (MVPA, recreational screen time, and sleep time) were included in a longitudinal design.

This study was expected to recruit a sample of Chinese-Australian adolescents from language schools, aged 12 to 17 years, living in Sydney, Melbourne, and Brisbane, Australia, for a six-month-period research investigation conducted from 2023 to 2024. Participants completed a set of self-reported questionnaires. The researchers visited four Chinese language schools several times. In order to implement a reliable study and encourage the participants to answer truthfully, the researchers presented and explained the study’s purpose, methodology, and questionnaires to the schools, parents/guardians, and students. The schools, parents/guardians, and students were empowered to give informed consent through clear communication and information. Trained research assistants administered the anonymous questionnaire, ensuring participants that their teachers could not access their responses. The questionnaire comprises well-established questionnaires that are effectively used in many countries [21,22]. It gathers essential data on subjects’ basic information, MVPA, social media usage, sedentary behaviours, sleep patterns, and psychological well-being.

To assess psychological well-being outcome, the adolescents completed the World Health Organization-Five Well-Being Index (WHO-5), which comprised five questions measuring the frequency of feeling ‘cheerful’, ‘calm and relaxed’, ‘active and vigorous’, ‘woke up feeling fresh and rested’, and ‘filled with interesting things’ during the past two weeks. Response options included ‘none of the time’ (0), ‘some of the time’ (1), ‘less than half the time’ (2), ‘more than half the time’ (3), ‘most of the time’ (4), and ‘all of the time’ (5). The range of scores for WHO-5 was thus from 0 to 25. A total score of 0–13 suggests poor well-being and may indicate the need for further evaluation or intervention. A total score of 14–17 indicates moderate well-being and suggests that the individual is experiencing some emotional distress. A total score of 18–25 suggests good well-being, indicating that the individual is likely experiencing a high level of emotional well-being and quality of life [21].

To measure participants’ physical activity, we asked them to report the number of physical education class hours they had on a typical school day, the total weekly hours spent playing team sports, and the total weekly hours dedicated to non-team sports at school.

Meeting the PA recommendation of 60 min of moderate-to-vigorous physical activity (MVPA) per day for seven days was dichotomized as either meeting or not meeting the recommendation.

Recreational screen time was assessed by asking participants about their daily hours of social media use, TV watching, video watching, and computer use for fun on both weekdays and weekends. The average daily screen time was calculated using a weighted formula: [5 × weekday screen time + 2 × weekend day screen time]/7. According to the Australian guidelines, meeting the screen time recommendation meant having no more than 2 h of recreational screen time per day for children and adolescents aged 5–17.

Sleep duration was assessed by asking participants about their typical sleep onset and wake-up times on school and non-school days and their average hours of sleep per day during weekdays and weekends. Based on Australian recommendations, meeting the sleep recommendation of 8–10 h for those aged 14–17 and 9–11 h for those aged 12–14 was determined.

The study considered age, measured in years, and gender (categorized as male, female, or other) as covariates. Adolescents self-reported their height (in centimetres) and weight (in kilograms). Based on these measurements, we calculated the body mass index (BMI) and incorporated it as a continuous variable in the analyses.

Descriptive variables were summarized using means and standard deviations for continuous variables and percentages for categorical variables. We calculated the proportions of meeting individual and combinations of recommendations within the Australian 24-HMGs. Linear regression models, adjusted for age, gender, and BMI, examined the associations between meeting specific recommendations and their combinations with psychological well-being. Due to the small percentage of adolescents meeting all three recommendations (1.2%), meeting three recommendations was merged with meeting any two for modelling purposes. Positive regression coefficients indicate better well-being, and negative coefficients suggest poorer well-being than the reference group. The data were analysed using Stata SE 17.0.

## 3. Results

During the implementation of the study, the researchers found a total of seven language schools. After making contact and conducting interviews, four schools responded. However, following further discussions and interviews, only two schools agreed to participate, while the others ceased responding despite our continued efforts to communicate. Although only two schools agreed to participate, one of them is the second-largest Chinese language school in Australia. Therefore, we believe the sample remains representative to a certain extent. During the recruitment process, 277 participants were recruited from two Chinese language schools. After evaluation, individuals outside the age range, unwilling to participate, or invalid survey data were excluded. There were 251 valid questionnaires for analysis. The survey was available in English and Chinese and took approximately 5–10 min to complete. Researchers collected data for this study from January to June 2023. Of the 600 surveys distributed, 277 students responded (46.1%). The Ethics Review Committee approved the study at the University of Queensland, Australia.

A total of 251 participants and 27 excluded individuals were collected, with no significant differences in sociodemographic variables, including gender, age, and BMI. Table 1 provides descriptive characteristics of the sample. The participants ranged from 12 to 13 years old, with 57.8% being female. The mean BMI was 19.61 (SD = 2.47), and the mean WHO-5 psychological well-being score was 13.69. Among the participants, 41% had WHO-5 scores below 13, indicating lower levels of psychological well-being. On average, they had 7.59 h of sleep per night. The average daily screen time was 2.06 h, occurring 5.2 days per week. They engaged in moderate-to-vigorous physical activity (MVPA) for an average of 2.26 days per week.

**Figure 1:**
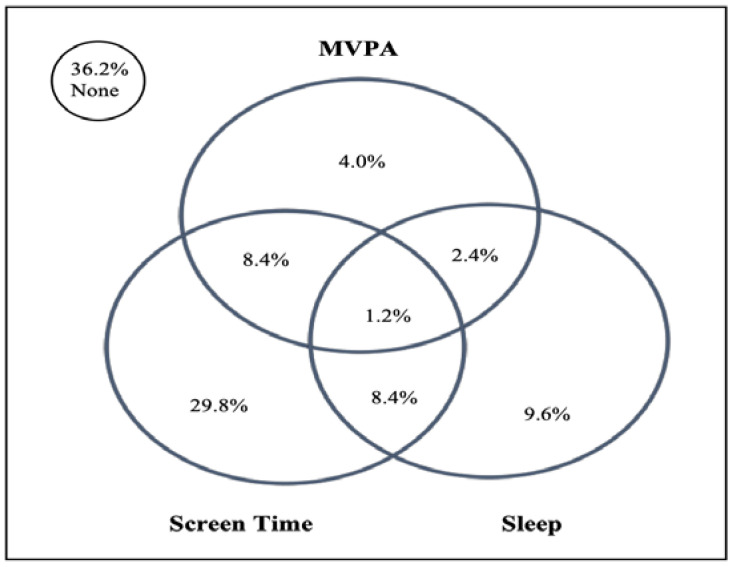
Venn diagram showing the proportions (%) of adolescents meeting the Australian 24-Hour Movement Guideline recommendations and no recommendation (*n* = 251).

Figure 1 presents the proportions of adolescents meeting specific and general combinations of recommendations. Over one-third (36%) still need to meet recommendations. Approximately 43% of the adolescents met at least one recommendation, while 19.1% met at least two. Only 1.2% of the participants met all three recommendations. Among those who met only one recommendation, meeting the screen time recommendation was the most prevalent (43%). Approximately 9.6% of the participants met the combined recommendations of MVPA and screen time or screen time and sleep. The multiple linear regression analysis indicated that psychological well-being was associated with meeting at least the MVPA recommendation (β = 1.41, 95% CI: 0.07 to 2.74) or at least the sleep recommendation (β = 1.40, 95% CI: 0.19 to 2.60) (Table 2). Meeting the combined recommendations of MVPA and sleep was significantly associated with higher psychological well-being (β = 3.83, 95% CI: 1.06–6.60). Moreover, achieving more recommendations (β = 1.68, 95% CI: 0.31 to 3.05) was significantly linked to improved psychological well-being compared to meeting only one recommendation (β = 0.56, 95% CI: −0.54 to 1.67).

## 4. Discussion

This study investigated the relationship between adherence to the 24-HMGs and psychological well-being among Chinese-Australian adolescents. The findings revealed that approximately 43% of adolescents adhered to at least one guideline, with screen time recommendation being the most followed, followed by MVPA and sleep recommendations. Psychological well-being was positively correlated with meeting either the MVPA or sleep recommendation, with significant improvement observed among those adhering to the combined MVPA and sleep recommendations (β = 3.83, 95% CI: 1.06–6.60). However, this study also highlighted a low compliance rate, with only 1.2% of participants meeting all three recommendations, which could be attributed to academic pressure, excessive screen time, and cultural factors that overshadow the importance of physical activity and sleep. This finding underscores the complex interplay of socio-cultural influences on adolescents’ health behaviours.

Compared to previous studies, this research is noteworthy for emphasising the combined effect of physical activity and sleep on psychological well-being, a topic that has not been sufficiently explored in the prior literature, which predominantly focused on the individual impacts of physical activity or sleep. Chinese-Australian adolescents displayed a significantly lower adherence rate to the 24-HMGs compared to their peers in Western countries, with academic pressure and family expectations being key contributing factors. In Chinese culture, academic excellence is highly valued, often at the expense of physical activity and rest. This aligns with previous findings, which showed that academic pressure is one of the main reasons adolescents prioritise study over exercise and sleep [23,24,25]; it can be observed that the scores for physical activity, sedentary behaviour, school-related support, and family support were ranked as low grades in the AHKGA Global Matrix. Furthermore, this study indicated that parental attitudes and family dynamics play a critical role in influencing adherence to the 24-HMGs. Strict parenting styles, especially those limiting outdoor activities and social interactions, reduce opportunities for physical activity and increase sedentary behaviour, negatively impacting both psychological and physical health [26,27]. The lack of adequate policy support and resources in the Chinese community further limits the promotion of physical activity. Although schools and communities can provide sports facilities and activities, the focus remains heavily on academic performance, leaving little room for promoting physical activities [28]. This cultural emphasis on academic success significantly reduces adherence to physical activity and sleep guidelines, making Chinese adolescents less likely to follow the 24-HMGs than their Western counterparts [24,25].

Previous studies have primarily focused on the individual effects of physical activity or sleep on psychological well-being, with fewer investigations examining their combined impact [9,29,30,31]. This study underscores the synergistic effects of both behaviours. Research has demonstrated that physical activity enhances neurotransmitters such as endorphins, reduces the stress hormone cortisol, effectively alleviates anxiety and depressive symptoms, improves self-efficacy, and boosts self-esteem [16,17,32]. Similarly, good sleep quality helps regulate emotional fluctuations and enhances emotional stability [29,30]. Sleep directly impacts the brain’s emotional processing regions, such as the amygdala, supporting memory consolidation and neuroplasticity. When adolescents receive adequate sleep, their attention, memory, and learning abilities improve, which helps mitigate academic stress-related anxiety and depression. Well-rested adolescents are also better equipped to handle stress and regulate negative emotions [19,33]. Conversely, sleep deprivation intensifies emotional reactivity, making adolescents more prone to depression, anxiety, or emotional overreactions [34]. While physical activity and sleep each have positive effects on adolescent psychological well-being, their combined influence significantly enhances these benefits [18]. This study suggests that such an integrated approach provides more long-term and comprehensive mental health benefits, particularly in stress management and emotional regulation, which are crucial for adolescent development.

This research also revealed that approximately 9.6% of participants adhered to the combined recommendations of MVPA and screen time or screen time and sleep. This finding emphasises the significant impact of balancing physical activity and reduced screen time on adolescents’ psychological well-being. Previous studies have shown that participating in sports or team activities not only enhances social support but also helps reduce feelings of loneliness and social disconnection [27,35]. This study highlights the importance of physical activity in establishing social connections, which are essential for reducing anxiety, depression, and emotional distress [36]. However, excessive screen time exacerbates social isolation by limiting face-to-face interactions, thus worsening psychological well-being. Unlike prior research that predominantly focused on social interaction, this study also considered the interaction between exercise, sleep, and screen time, offering a more holistic view of adolescents’ psychological well-being.

This research provides new insights into the application of the 24-HMGs among Chinese-Australian adolescents and highlights the need for future interventions to focus on maintaining physical activity and sleep habits while reducing screen time, particularly in the context of academic pressure. Promoting better social support systems and improving mental health outcomes for adolescents requires a comprehensive approach that addresses the interconnectedness of these behaviours. The following recommendations aim to provide practical measures for schools and parents to improve physical activity, sleep, and screen time behaviours, with the goal of helping public health organisations and policymakers develop effective strategies to enhance adolescent psychological well-being. It is recommended that parents actively encourage their children to engage in physical activity, given the long-term positive effects of healthy behaviours on mental health. Furthermore, families and schools should establish reasonable screen time limits, utilizing digital tools such as health tracking applications to help children manage their health behaviours. These tools can assist students in balancing academic, physical, and rest time, reducing the negative impacts of excessive screen time, and promoting overall well-being [26,27]. Schools should increase the availability of multifunctional sports facilities and provide open hours for students to engage in physical activities during after-school hours or vacations, reducing sedentary behaviour. Special attention should be given to groups with higher academic pressures, helping them to understand the positive role of physical activity in psychological well-being [28,37]. Most importantly, adolescents must be seen as the central target group of this strategy, helping them to learn to manage their health behaviours such as physical activity, diet, and sleep. In summary, providing support from both schools and parents is crucial in creating a resource-rich and supportive environment that aids adolescents in changing unhealthy behaviours, improving their mental health, and reducing the risks of anxiety, depression, and suicide.

### Limitations

Despite the fact that our study provides valuable insights, there are some limitations which may influence the interpretation and generalisability of the results. First, the cross-sectional design of this study limits the ability to determine causal relationships. Since the data were collected at a single point in time, it was not possible to establish whether the three behaviours directly contributed to improvements in psychological well-being. Future research should employ longitudinal designs to explore these behaviours and their relationship with psychological well-being, providing a clearer understanding of their chronological order and cause-and-effect dynamics. Secondly, there are limitations regarding the sample size. Although the sample was collected from two Chinese language schools in Brisbane, it may not be able to represent the broader youth population across Australia or diverse socioeconomic backgrounds. Future research should expand the sample to include individuals from different cities and socioeconomic backgrounds to enhance the external validity of the findings. Third, this study used self-report questionnaires to collect data from participants. While this method is simple and efficient, its validity and reliability remain open to discussion. Self-reported data are susceptible to social desirability bias, where participants may report behaviours that they perceive as socially desirable rather than their actual behaviours. Future research should consider using wearable devices to track physical activity and sleep time, improving data accuracy and reliability.

## 5. Conclusions

This study highlights the significant impact of physical activity, sleep, and screen time on the psychological well-being of Chinese-Australian adolescents. The findings demonstrate that adolescents who adhere to both physical activity and sleep recommendations show significant improvements in psychological well-being.

## Figures and Tables

**Table 1:** Description of study sample from Chinese Language Schools (*n* = 251).

Characteristics	All Sample
Age, years [M (SD)]	13.31 (1.34)
Age range, years	12–13
Body mass index (BMI)	19.61 (2.47)
Girl [%]	57.8%
Movement behaviours [M (SD)]	
Average MVPA (days/week)	2.26 (1.91)
Classroom time (h)	5.04 (0.65)
PE time (h)	1.03 (0.61)
Team sports time (h)	1.29 (2.08)
Non team sports time (h)	0.78 (1.01)
Average Psychological well-being	13.69 (4.46)
Under 13 (%)	41.04%
Average Sleep Time, h/night	7.59 (1.13)
Average screen time, h/day	2.06 (1.94)
Average screen time, day/weeks	5.2 (2.48)

**Table 2:** Associations between meeting the physical activity, screen time, and sleep recommendations and combinations of these recommendations with psychological well-being among Chinese Australian adolescents (*n* = 251).

Recommendations	%	Wellbeing	*p*-Value *
Specific combinations		β (95% CI) *	
Meeting at least MVPA	15.9	1.41 (0.07 to 2.74)	0.039 *
Meeting at least ST	47.8	0.24 (−0.75 to 1.23)	0.63
Meeting at least sleep	21.5	1.40 (0.19 to 2.60)	0.023 *
Meeting both MVPA + ST	9.6	0.30 (−1.37 to 1.98)	0.722
Meeting both MVPA + sleep	3.6	3.83 (1.06 to 6.60)	0.007 *
Meeting both ST + sleep	9.6	1.38 (−0.42 to 3.18)	0.133
General combinations			
None	36.2		
One out of three	43.3	0.56 (−0.54 to 1.67)	0.314
Two/three out of three	20.3	1.68 (0.31 to 3.05)	0.016 *

* *p* < 0.05.

## Data Availability

The original contributions presented in the study are included in the article, further inquiries can be directed to the corresponding author.
